# Supplementary material for: Screen-Printed Glucose Sensors Modified with Cellulose Nanocrystals (CNCs) for Cell Culture Monitoring
Source: Biosensors (Basel). 2020 Sep 13;10(9):125. doi: 10.3390/bios10090125 (PMC7557574; doi:10.3390/bios10090125)
Supplement: Supplementary file 1 [file biosensors-10-00125-s001.pdf]

biosensors

Supplementary information

Screen-printed glucose sensors for cell culture monitoring modified by cellulose nanocrystals (CNCs)

Ye Tang [1,2], Konstantinos Petropoulos [1], Felix Kurth [1], Hui Chai-Gao [1], Davide Migliorelli [1], Olivier Guenat [2] and Silvia Generelli [1,*]

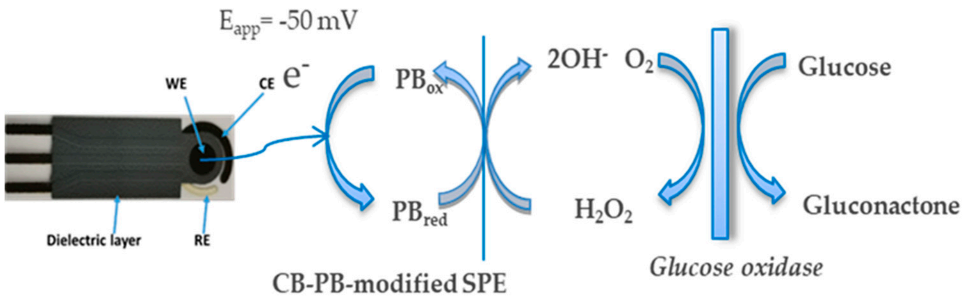

Figure S1. Schematic view of the sensor working principle. WE: working electrode, RE: reference electrode, CE: counter electrode.

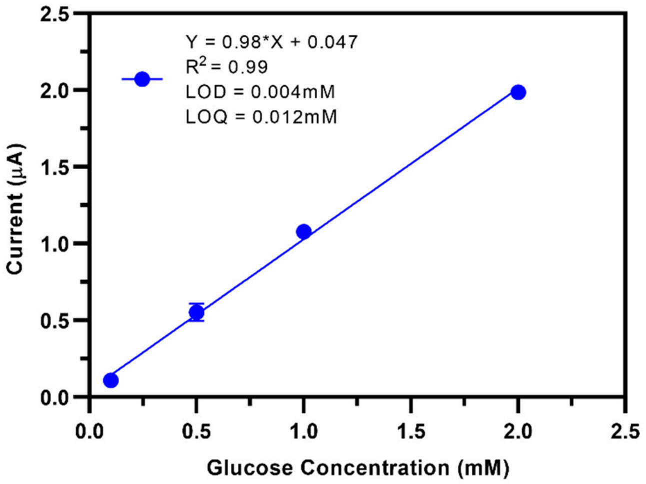

Figure S2. Calibration curve of the glutaraldehyde-based glucose sensor (GA sensor) in 0.1–2 mM glucose in phosphate buffer. The linear range (up to 2 mM) was smaller than the entire dynamic range. n = 3; plotted are the mean ± standard deviation and the linear fit from 0.1 to 2 mM.

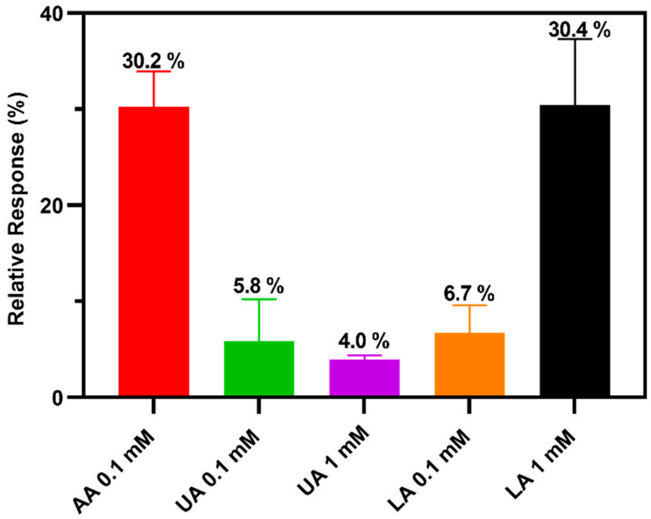

Figure S3. Relative responses of the GA sensor toward interfering species (0.1 mM ascorbic acid, 0.1 and 1 mM uric acid, 0.1 and 1 mM LA) in a 1 mM glucose standard solution in phosphate buffer. n = 3 for each condition; 1 mM glucose without interfering species is defined as 100 % response.

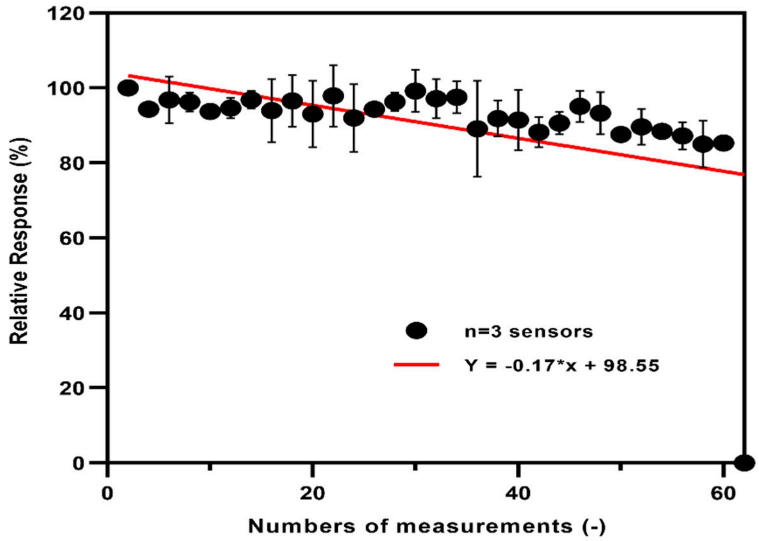

Figure S4. Operational stability of the GA sensor over 30 repeated measurements in 1 mM glucose solution in phosphate buffer. The graph depicts the average values of three individual sensors (mean ± standard deviation); the red linear fit indicates the loss in measured current over the 30 cycles (total of 6 hours of measurement). The average remaining activity of the TEMPO-CNC glucose sensor after 30 measurements was 85.3 % of the initial current.

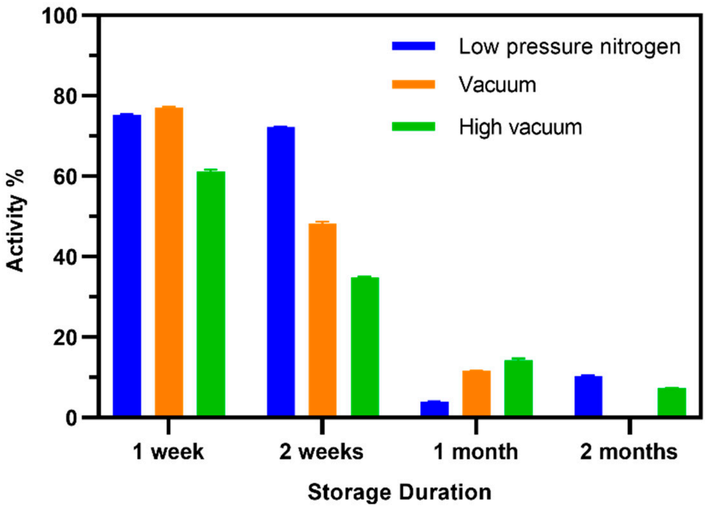

Figure S5. Shelf-life test of the GA sensor under different storage conditions: low-pressure nitrogen (400 mbar N_2), vacuum (≤ 50 mbar), and high-vacuum (≤ 10 mbar) drying treatment before vacuum packing. All sensors were stored at 4 °C. The sensors were tested after 1 week, 2 weeks, 1 month, and 2 months. All values are normalized against the signals of sensors tested without storage.

Table S1. TEMPO-CNCs glucose sensor performance (response time, sensitivity, linear range, limit of detection, reproducibility).

**TEMPO-CNC glucose sensor**	
**Response time (s)**	30
Sensitivity (μA·cm^{-2}·mM^{-1})	5.7
Linear Range [mM]	0.1-2.0
Limit of detection [mM]	0.004
Limit of quantification [mM]	0.015
Reproducibility RSD (%)	4.6

Table S2. GA sensor performance (response time, sensitivity, linear range, limit of detection, reproducibility).

**GA sensor.**	
**Response time (s)**	30
Sensitivity (μA·cm^{-2}·mM^{-1})	14.1
Linear Range [mM]	0.1-2
Limit of detection [mM]	0.004
Limit of quantification [mM]	0.012
Reproducibility RSD (%)	10.2
